# Resveratrol Content Profiles and Their Correlation with Multidimensional Quality in Different Peanut Cultivars

**DOI:** 10.3390/foods15071172

**Published:** 2026-03-31

**Authors:** Yumeng Hu, Jiaxin Guo, Tian Li, Mengjiao Zhang, Zefang Jiang, Qiang Wang, Qin Guo

**Affiliations:** 1 Institute of Food Science and Technology, Chinese Academy of Agricultural Sciences, Key Laboratory of Agro-Products Processing, Ministry of Agriculture, Beijing 100193, China; 2 Key Laboratory of Food Nutrition and Functional Food of Hainan Province, School of Food Science and Engineering, Hainan University, Haikou 570228, China

**Keywords:** peanut cultivars, resveratrol, resveratrol glycoside, quality indicators, correlation analysis

## Abstract

Resveratrol is a promising polyphenolic bioactive compound found in peanuts. However, the distribution of *cis*- and *trans*-resveratrol and their glycosides varies significantly among cultivars, and their correlations with other quality traits remain unclear. In this study, Ultra-Performance Liquid Chromatography (UPLC) combined with high-throughput spectral analysis was employed to systematically evaluate 42 main cultivated peanut varieties from seven series across China’s three major production regions. The results indicated that *trans*-piceid (*trans*-resveratrol glycoside) was the predominant component, accounting for over 85% of the total content. Significant variation was observed in the total content of resveratrol and its glycosides among cultivars (4.61–88.79 mg/kg), with the Weihua series (represented by Weihua 23) exhibiting the strongest resveratrol enrichment ability. Multidimensional correlation analysis systematically revealed, for the first time, distinct association patterns: *trans*-piceid was positively correlated with sucrose, *cis*-resveratrol was positively correlated with fatty acids such as oleic acid, and *trans*-resveratrol showed a specific association with linoleic acid. Based on these findings, seven specialized high-resveratrol cultivars suitable for processing, including Weihua 23 and Kainong 301, were identified. Furthermore, a specific correlation system between “resveratrol and key quality indicators” was established. This study provides important theoretical support for the targeted breeding of novel functional peanut varieties that combine a high resveratrol content with traits such as high oleic acid or sugar content, thereby promoting the high-value industrial utilization of high-quality peanuts.

## 1. Introduction

Peanuts are a characteristic agricultural product of China and possess a significant competitive advantage in the international market [1]. The country maintains an absolute global lead, ranking first in four key categories: total production, total processing output value, total import and export volume, and oil yield; in 2024, China’s total peanut production reached 19.00 million tons, accounting for 38.8% of the global total [2]. A deep excavation of the nutritional and functional value of peanuts, and consequently, an in-depth exploration of their nutritional and functional value, particularly the systematic elucidation of their bioactive components, is of great significance for promoting the healthy upgrading of the peanut food industry.

Resveratrol, a highly promising polyphenolic bioactive substance found in peanuts [3], exhibits various physiological functions, including anticancer [4], cardiovascular protection [5], and anti-inflammatory effects [6]. Resveratrol has received considerable attention from scientists in recent years due to these bioactive properties. Peanut and peanut-derived products are recognized as natural sources of resveratrol. Previous studies have shown that the content of resveratrol in peanuts is influenced by genotype [7], processing [8], germination [9], and elicitation treatments [10]. However, most available studies have focused on *trans*-resveratrol, processing-induced changes [11], or specific peanut products such as peanut butter and peanut oil. While it has become a core ingredient in the development of functional foods [12], the characteristics of its content profiles in peanuts and their correlation with multidimensional quality attributes have not yet been systematically investigated.

Although China possesses over 8900 peanut germplasm resources and more than 300 main cultivars [13], there are significant differences in the contents of resveratrol, its *trans*-/*cis*-isomers, and its glycoside derivatives across different regions and varieties. Wang [14] found that the resveratrol content in peanuts showed a gradient trend of Liaoning being higher than Henan, and Henan being higher than Shandong. However, existing research has primarily focused on the detection of total resveratrol content, lacking a systematic analysis of its isomers and glycoside derivatives. Furthermore, the synergistic effects of resveratrol and core quality indicators, such as proteins and fatty acids, remain unclear, which limits the screening of high-content germplasms and the development of related products.

This study systematically quantifies the content of *cis*-/*trans*-resveratrol and its glycoside derivatives in 42 widely cultivated peanut varieties from China’s four major production regions. We simultaneously analyzed the physicochemical compositions (e.g., fat, protein, and sucrose) and multidimensional quality indicators (e.g., fatty acids, amino acids, and protein fractions). By establishing a quantitative relationship network between “bioactive components and quality indicators,” this study aims to clarify the distribution patterns of resveratrol content across different regions and varieties, as well as its synergistic interactions with core quality traits, thereby providing theoretical support for high-quality peanut breeding and the development of nutritional functional products.

## 2. Materials and Methods

### 2.1. Materials and Reagents

In this study, 42 peanut cultivars from 7 series were selected as samples (Table 1). These series represent the major breeding backgrounds and widely cultivated cultivar groups in China, covering different genetic characteristics and ecological adaptability. All selected cultivars are state-certified and have been widely promoted in the major peanut-producing regions of China, with planting areas exceeding 300,000 mu (approximately 20,000 ha), ensuring the broad representativeness of the research materials. All samples were collected in September 2024. Samples were stored at 4 °C and analyzed within three weeks after harvest.

All reagents used in the experiment were of high-purity chromatographic grade. HPLC-grade ethanol was obtained from Merck KGaA (Darmstadt, Germany). HPLC-grade formic acid was provided by Sepure Scientific (Beijing) Technology Co., Ltd. (Beijing, China). HPLC-grade methanol was acquired from Thermo Fisher Scientific Inc. (Waltham, MA, USA). Trans-resveratrol standard (purity ≥ 98%, HPLC) was purchased from Beijing Solarbio Science & Technology Co., Ltd. (Beijing, China). *Trans*-resveratrol glucoside (polydatin, purity ≥ 98%, HPLC) standard was supplied by Shanghai Yuanye Bio-Technology Co., Ltd. (Shanghai, China). Cis-resveratrol standard (purity ≥ 98%, HPLC) was obtained from Sichuan Weikeqi Biological Technology Co., Ltd. (Chengdu, Sichuan, China). Additionally, *cis*-resveratrol glycoside (*cis*-piceid) was prepared by irradiating *trans*-resveratrol glycoside with 365 nm ultraviolet (UV) light. Portable high-throughput peanut quality analyzer (Beijing Kaiyuan Hongyu Technology Co., Ltd., Beijing, China). Ultra-high-performance liquid chromatograph (Waters Corporation, Milford, MA, USA). Chromatography column (Acquity UPLC HSS C18, 1.8 μm, 2.1 × 100 mm, Waters Corporation, Milford, MA, USA).

### 2.2. Determination of Resveratrol Content

Peanut kernel samples (with red skins, 1.0 g) were weighed and ground to pass through a 40-mesh sieve. The powder was mixed with 8 mL of 80% ethanol (*v*/*v*) [15], vortexed for 5 min, and centrifuged at 5000 r/min for 10 min. The extraction process was repeated thrice. The supernatants were combined, transferred to a 25 mL volumetric flask for volume adjustment, and filtered through a 0.22 μm organic membrane filter before analysis. Liquid chromatography analysis was performed using a C18 column (2.1 mm × 100 mm, 1.8 μm) maintained at 35 °C. The detection wavelength was set at 306 nm, the injection volume was 10 μL, and the flow rate was 0.45 mL/min. The mobile phases consisted of 0.1% aqueous formic acid (A) and pure methanol (B). Gradient elution was applied as follows: 0–0.5 min, 90% A; 0.5–2.0 min, 75–90% A; 2.0–3.5 min, 70–75% A; 3.5–4.0 min, 65–70% A; 4.0–5.0 min, 50–65% A; 5.0–7.0 min, 10–50% A; 7.0–7.2 min, 10–90% A; and 7.2–10.0 min, 90% A [15].

### 2.3. Determination of Quality Indicators

All 42 peanut samples were prepared for quality trait determination. Spectral data acquisition for the different peanut cultivars was performed using a portable high-throughput peanut quality analyzer developed in-house by the Institute of Food Science and Technology, Chinese Academy of Agricultural Sciences (CAAS) [16,17]. Peanut kernels (with red skins, 30–50 g) were placed into the sample cup, filling it to approximately one-quarter of its height. Following successful calibration, the sample cup was positioned in the detection zone and scanned thrice. The contents of various quality indicators, including moisture, fat, protein and protein fractions, sucrose, amino acids, and fatty acids, were quantified using previously established spectral models. The instrument has been successfully applied in previous studies for the determination of the above indicators [18,19,20,21]. All measurements were performed in triplicate, and average values were calculated.

### 2.4. Data Analysis

Data processing and statistical analyses were performed using Microsoft Excel and SPSS 27 software. Figures were generated using Origin 2021. All experiments were performed with at least three independent replicates, and the results are presented as mean ± standard deviation (SD). Prior to analysis, the data were checked for completeness and screened for obvious outliers. Differences among groups were analyzed by one-way analysis of variance (ANOVA), followed by Tukey’s multiple comparison test. Correlation analysis was performed using Pearson’s correlation coefficient. A value of *p* < 0.05 was considered statistically significant.

## 3. Results and Discussion

### 3.1. Analysis of Resveratrol Content in Different Peanut Cultivars

This study analyzed the resveratrol content of 42 peanut cultivars representing seven series from four major production regions in China (Table 2).

**Figure 1:**
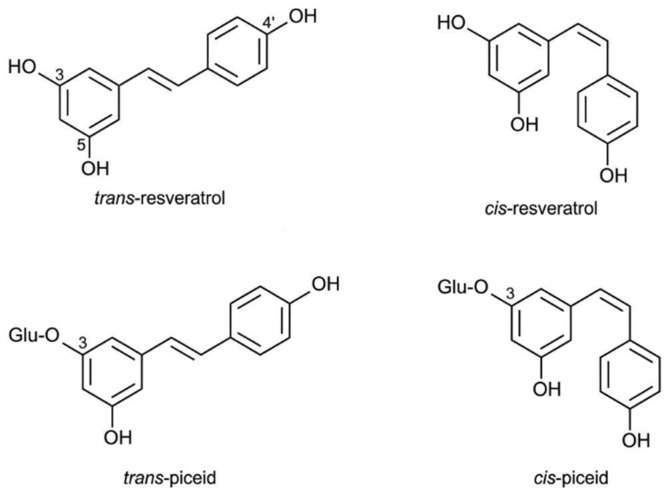
Molecular structures of resveratrol and resveratrol glycoside isomers.

The results indicated that resveratrol in peanuts primarily exists as *trans*-resveratrol, *trans*-piceid (resveratrol glycoside), and *cis*-resveratrol. *Cis*-piceid was not detected in any samples, and the content of *trans*-piceid was significantly higher than that of both *cis*- and *trans*-resveratrol. Figure 1 shows the molecular structures of resveratrol and resveratrol glycoside isomers. The total content of resveratrol and its glycosides varied significantly among the cultivars (*p* < 0.05), ranging from 4.61 to 88.79 mg/kg, with a coefficient of variation (CV) of 47.66%. Specifically, *trans*-piceid was the predominant component, with an average content of 31.77 mg/kg, accounting for 87.1% of the total content. This was followed by *trans*-resveratrol (average content 3.36 mg/kg, 9.2%) and *cis*-resveratrol (average content 1.34 mg/kg, 3.7%). Notably, in all samples from the Yuhua series, the content of *cis*-resveratrol was below the limit of detection (LOD) and could not be quantified.

Significant differences have been observed in the resveratrol content among different peanut series [22]. The Huayu and Kainong series, primarily produced in the Shandong and Henan provinces, exhibited the best performance, serving as high-quality germplasm resources for breeding high-resveratrol varieties. Specifically, the Weihua series from the Shandong production region ranked first among all series, with an average *trans*-piceid content of 50.82 mg/kg and an average total resveratrol content of 54.47 mg/kg. Notably, the cultivar Weihua 23 reached a total content of 88.79 mg/kg, which was 2.43 times the overall average. Conversely, the Kainong series demonstrated an advantage in *cis*-resveratrol content, with an average of 3.38 mg/kg (2.52 times the overall average). Specifically, Kainong 1760 reached a *cis*-resveratrol content of 11.47 mg/kg. Additionally, Kainong 602, Kainong 603, and Yuhua 9326 exhibited relatively high *trans*-piceid contents, whereas the Puhua series had an average of 22.95 mg/kg. These findings highlight the synergistic advantage of the Yellow River Basin (Shandong and Henan) region for screening high-resveratrol germplasm.

Although high-resveratrol cultivars exist in the remaining series, their overall content shows high variation and scattered distribution. It is worth emphasizing that the resveratrol-enriched cultivars identified in this study, such as Weihua 23 and 22, and Kainong 1760, 602, and 603, provide a scientific basis and high-quality raw materials for the targeted breeding of high-resveratrol peanut varieties and the development of functional foods.

### 3.2. Analysis of Quality Traits in Different Peanut Cultivars

#### 3.2.1. Moisture Content

Significant differences have been observed in the moisture content of different peanut series under identical drying temperatures and durations [23]. As shown in Figure 1, the moisture content of the peanut kernels ranged from 3.10 to 6.79 g/100 g, with an overall coefficient of variation (CV) of 14.55%. The Huayu series, primarily produced in Shandong, exhibited the highest average moisture content of 5.60 g/100 g. Among all individual samples, Weihua 22 recorded the highest value among all samples at 6.79 g/100 g. Conversely, the Kainong series, primarily bred in Henan, had the lowest average moisture content of 4.77 g/100 g, with Kainong 601 recording the lowest individual value at 3.90 g/100 g. These discrepancies may be attributed to two factors. First, genetic variations among cultivars result in differentiated regulatory mechanisms for moisture absorption, retention, and loss during growth and development, leading to inherent differences in seed moisture content at maturity [24,25]. Second, the climatic environment of the production region exerts a significant influence. The relatively humid and rainy climate of the Shandong region promotes greater moisture absorption during growth, whereas the relatively dry climate of the Henan region (excluding years with natural disasters) facilitates faster moisture loss during both growth and drying stages [26,27].

#### 3.2.2. Crude Fat Content

The fat content of peanut kernels is a critical factor in determining their economic value. As shown in Figure 1, the fat content across different peanut cultivars ranged from 46.48 to 53.09 g/100 g, with a coefficient of variation (CV) of 3.41%. In terms of series, the Huayu series (primarily produced in Shandong) exhibited the highest average crude fat content, reaching 50.73 g/100 g, while the Weihua series had the lowest average content (with Weihua 23 recording 48.46 g/100 g). At the cultivar level, Kainong 1715 from Henan demonstrated a crude fat content as high as 53.09 g/100 g, which is 5.72% higher than the overall average, making it an excellent cultivar for oil production. Analysis [28] of 688 peanut cultivars from 22 provinces across China (covering all series in this study) showed that there were no significant differences in peanut fat content at the provincial level except for Liaoning and Shandong. However, Guo et al. [29] analyzed six different peanut cultivars across four regions in Guangdong Province and found that the variation index for fat content in the same cultivar grown across different regions could reach 12.5%. This variation, likely caused by significant genotype-by-environment (G × E) interactions, far exceeded the differences observed between cultivars within the same region. This suggests that at the microscale, genetic and environmental factors are key determinants of fat content variation, whereas at the macroscale, cultivar diversity dilutes the differences in fat content between regions.

#### 3.2.3. Protein Content

The protein content of peanut kernels is a core indicator for evaluating the nutritional quality of peanuts [30]. As shown in Figure 1, the protein content of different peanut series ranged from 18.77 to 30.72 g/100 g, with a coefficient of variation (CV) of 12.31%, indicating significant differences among cultivars. Among these series, the Yuhua series (primarily produced in Henan) exhibited the highest average protein content (26.68 g/100 g). Both the Yuhua and Kainong series demonstrated relatively low inter-cultivar variation, suggesting a stable quality. Specifically, Yuhua 23 (30.40 g/100 g) was identified as a superior high-protein germplasm. The Huayu series from the Shandong production region had an average protein content of 24.07 g/100 g. Although this average was lower than that of the Yuhua series, the Huayu series contained superior high-protein germplasms, namely Huayu 33 (30.34 g/100 g) and Huayu 36 (30.19 g/100 g). Among all the cultivars, Weihua 27 exhibited the most outstanding performance, with a protein content of 30.72 g/100 g. This characteristic endows Weihua 27 with immense potential for use in high-protein food ingredient development and plant protein extraction, making it an excellent candidate for cultivar selection.

#### 3.2.4. Sucrose Content

**Figure 2:**
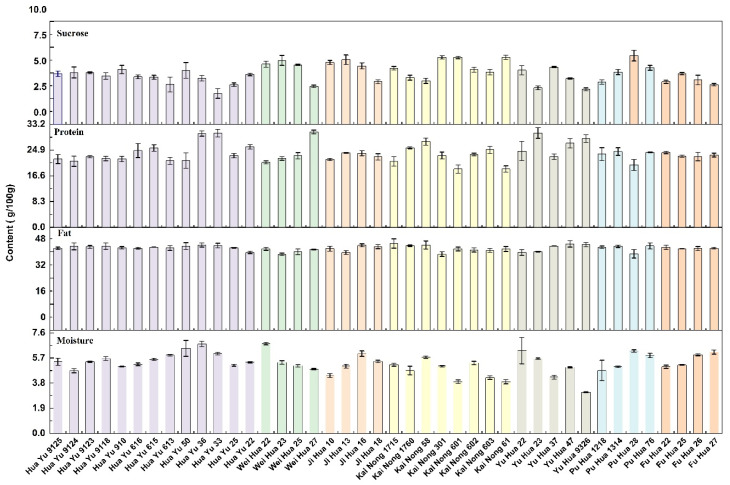
Moisture, fat, protein and sucrose contents of different peanut cultivars.

Sucrose is a critical quality indicator that influences the flavor and sweetness of roasted peanuts [31]. As shown in Figure 2, the sucrose content across different peanut cultivars ranged from 3.00 to 6.68 g/100 g, with a coefficient of variation (CV) of 18.77%, indicating significant variability. Among the series, the Jihua series (primarily produced in Hebei) exhibited the highest average sucrose content, reaching 5.53 g/100 g. The Weihua series from the Shandong production region and the Kainong series from Henan showed similar distributions, both with an average sucrose content of 5.30 g/100 g. The sucrose content of the remaining cultivars was relatively low. At the individual cultivar level, Puhua 28 reached a peak value of 6.68 g/100 g, exceeding the overall average by 34.95%. Kainong 301 (6.51 g/100 g) and Kainong 601 (6.50 g/100 g) also demonstrated excellent sucrose accumulation. These findings suggest that these cultivars possess a natural flavor advantage, making them highly suitable as raw materials for fresh consumption or roasting [32].

#### 3.2.5. Fatty Acid Composition

The fatty acid composition of peanuts is a core determinant of the nutritional quality, oxidative stability, and processing suitability of their oil [33], making it crucial for the development of functional oils. Significant functional differentiation in fatty acid composition was observed among peanuts from different production regions. Analysis of the 42 cultivars revealed considerable variation in fatty acid profiles: oleic acid (C18:1) content ranged from 34.09% to 79.92% (CV 25.74%), and linoleic acid (C18:2) content ranged from 3.15% to 47.87%. Other fatty acids also exhibited inter-cultivar variation (Table 3), including palmitic acid (C16:0, 5.11–12.93%, CV 26.66%), arachidic acid (C20:0, 0.74–1.61%, CV 17.47%), and stearic acid (C18:0, 0.98–3.57%, CV 3.53%).

Regarding oleic acid and unsaturated fatty acid content, the selected varieties from

Kainong series from Liaoning exhibited high and stable oleic acid levels, with an oleic/linoleic (O/L) ratio exceeding 25 (Kainong 1760). This characteristic improved the oxidative stability of the oil by 40%, significantly enhancing the processing stability and shelf life of products such as roasted peanuts and peanut butter. Meanwhile, the Weihua series from Shandong recorded the highest mean oleic acid content (65.2%), demonstrating significant potential for high oleic acid breeding. Among the individual cultivars, Kainong 1715 was the most outstanding, achieving an oleic acid content of 81.91%. Furthermore, Yuhua 22 and Kainong 1715 exhibited excellent performance in terms of total unsaturated fatty acids (94.71% and 93.84%, respectively), marking them as valuable genetic resources for developing functional nutritional foods.

#### 3.2.6. Amino Acid Composition

This study analyzed the compositions of 18 amino acids in different peanut series. These 18 amino acids include Histidine, Tryptophan, Methionine, Cysteine, Threonine, Valine, Isoleucine, Leucine, Phenylalanine, Lysine, Proline, Alanine, Serine, Tyrosine, Glycine, Glutamic Acid, and Aspartic Acid. As key flavor precursors, variations in the types and contents of these amino acids contribute significantly to the unique flavor characteristics of peanuts [34]. The total amino acid content of the 42 peanut cultivars ranged from 19.90 to 38.89 g/100 g. This included seven essential amino acids, with leucine being the most abundant, followed by phenylalanine, and methionine being the least abundant. Among the non-essential amino acids, glutamic acid was the predominant one, accounting for 10.36–24.56% of the total amino acid content. In terms of regional series, the Kainong series from Henan (represented by Kainong 58 and Kainong 176) was characterized by elevated serine, alanine, and tyrosine levels. These amino acids are typically associated with enhanced sweetness and umami, endowing the Kainong series with the potential to generate a richer and more balanced flavor profile. In contrast, the amino acid composition patterns of the Huayu series from Shandong (such as Huayu 25 and Huayu 36) were relatively consistent, providing a stable basis for raw material utilization. Among the individual cultivars, Kainong 58 also demonstrated significant potential for developing high-arginine (4.23%) peanut-based foods.

#### 3.2.7. Associations Between Peanut Resveratrol (and Glycosides) and Multidimensional Quality Indicators

**Figure 3:**
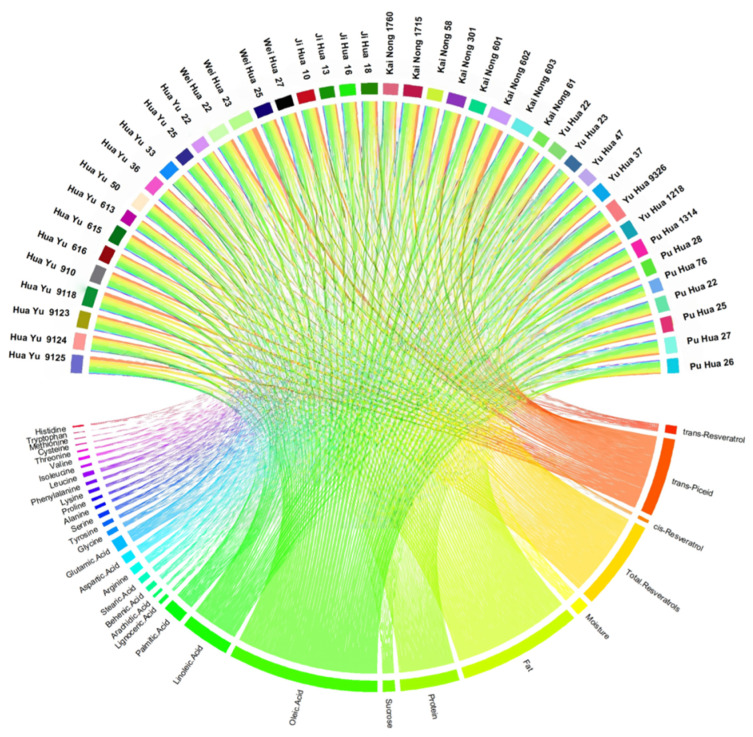
Chord diagram of quality indicators in different peanut cultivars.

To systematically summarize the observed associations between resveratrol (and its glycosides) and multidimensional quality indicators across different peanut cultivars, a correlation network chord diagram was constructed (Figure 3). In this diagram, the thickness of each chord visually represents the strength of the association between peanut cultivars and quality indicators, showcasing the unique correlation patterns of quality traits in different series of peanut varieties. The results indicated that Huayu 36, Kainong 1760, Kainong 602, and Weihua 23 showed comparatively higher levels of *trans*-resveratrol, *cis*-resveratrol, *trans*-piceid, and total resveratrol. This variation suggests cultivar-dependent patterns in the accumulation of resveratrol and its glycosides accumulation in peanut cultivars. This cultivar dependence may be related to differences in the stilbene biosynthetic pathway. For example, STS has been reported to participate in stilbene formation [35]. However, the present correlation analysis cannot determine whether STS activity causally drives the observed differences. Given their higher resveratrol-related metabolite levels, these cultivars may be considered as promising candidate parental materials for breeding programs, pending further genetic validation. Utilizing these cultivars provides a reference basis for the targeted breeding of high-resveratrol germplasm and can significantly enhance germplasm screening efficiency. Furthermore, network analysis revealed prominent associations between cultivars, such as Weihua 23, Jihua 16, and Jihua 18, and key fatty acids (e.g., oleic and linoleic acids). This pattern is consistent with the possibility of cultivar-specific regulation related to fatty acid metabolism. Genes such as FAD2 have been implicated in linoleic acid balance [36], but additional genotypic or transcriptomic data would be needed to verify this mechanism in our materials. These findings offer theoretical support for the development of specialized cultivars, such as high-oleic acid varieties, thereby meeting the demand for upgraded edible oil quality in the future.

**Figure 4:**
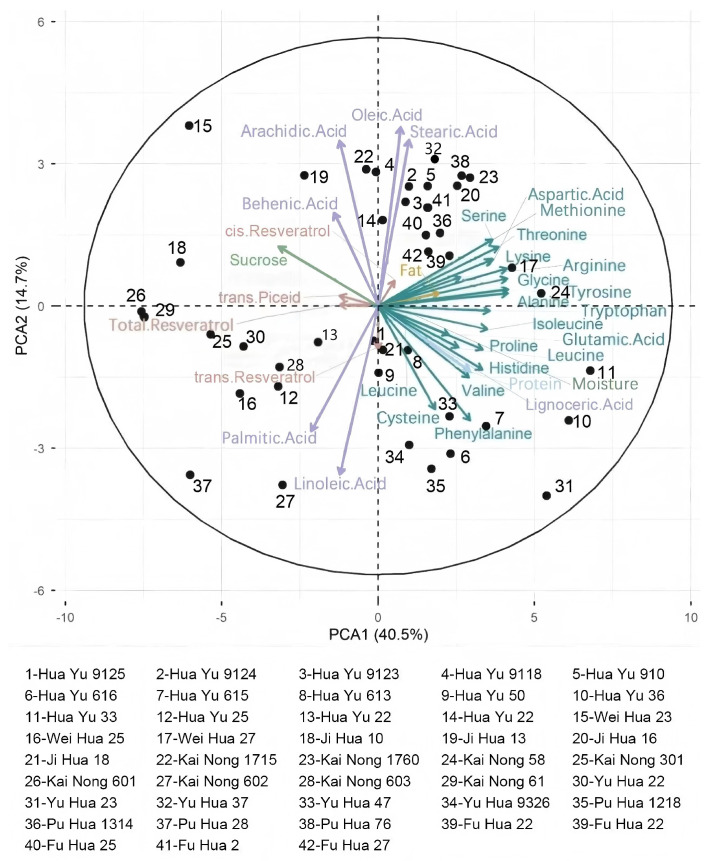
Principal Component Analysis of Quality Indicators of Different Peanut Varieties.

To explore multivariate association patterns among resveratrol-related traits and other quality indicators, and to facilitate a comprehensive evaluation and classification of peanut cultivars, principal component analysis (PCA) was performed (Figure 4). The first two principal components (PC1 and PC2) cumulatively explained 55.2% of the total variance, effectively capturing core information from the dataset. PC1 (explaining 40.5% of the variance) was characterized by high positive loadings of proteins and amino acids (such as arginine, glutamic acid, and lysine). In contrast, *trans*-piceid and total resveratrol exhibited distinct negative loadings. This opposite loading pattern is consistent with a potential trade-off between protein-related traits and resveratrol-related traits; however, PCA describes co-variation and does not establish a mechanistic competition in carbon–nitrogen allocation, which would require further physiological or molecular validation [37]. PC2 was primarily driven by fatty acid components, reflecting the oil composition, with oleic and linoleic acids exhibiting characteristic inverse distributions [38].

Specific correlation patterns were observed among the chemical components: *trans*-piceid exhibited a positive correlation with sucrose and behenic acid, *trans*-resveratrol was positively correlated with linoleic acid, palmitic acid, phenylalanine, and cysteine, and *cis*-resveratrol showed positive correlations with stearic acid, oleic acid, arachidic acid, and behenic acid. These co-variation patterns may indicate coordinated accumulation and shared regulation; however, dedicated pathway-level or transcriptomic analyses would be required to test whether common biosynthetic routes or synergistic interactions underlie these relationships. In the PCA biplot, cultivars such as Kainong 602, Kainong 603, Weihua 23, Huayu 9118, Weihua 22, Kainong 301, and Yuhua 9326 were positioned at the positive projection end of the vectors for the total resveratrol and *trans*-piceid. This positioning is generally consistent with the ranking of high-content varieties in the raw data, indicating a consistent accumulation trend of resveratrol in these cultivars. These findings provide a reference for screening high-resveratrol germplasm. Simultaneously, cultivars such as Huayu 9118, Kainong 301, and Kainong 1760 maintained relatively high levels of resveratrol and oleic acid. This suggests that these cultivars may be promising candidate donor materials for developing functional peanut varieties with combined high-oleic and high-resveratrol profiles, pending further breeding evaluation and genetic validation.

**Figure 5:**
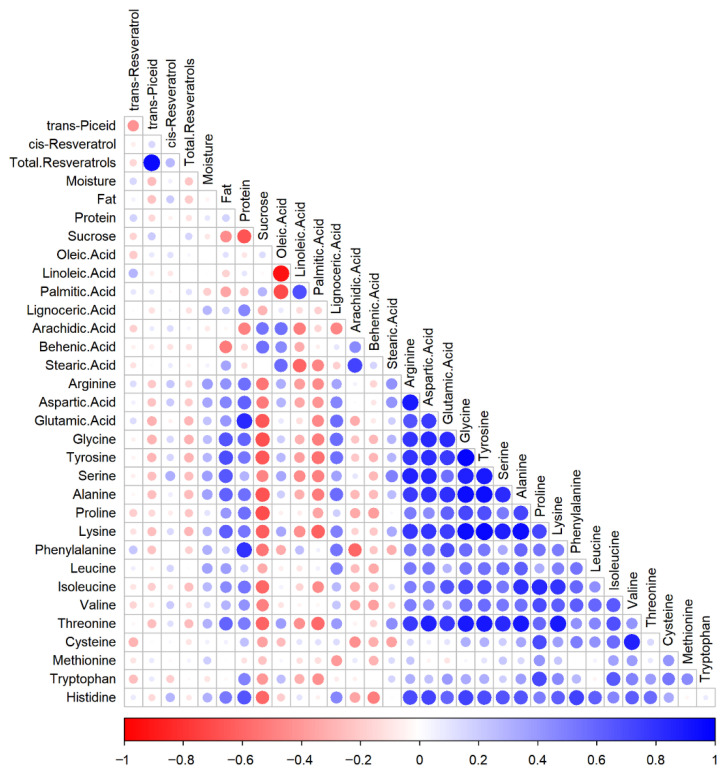
Correlation heatmap of quality indicators in different peanut cultivars.

The Pearson correlation heatmap (Figure 5) quantitatively summarized the correlations between resveratrol (and its glycosides) and various quality indicators, consistent with the chord diagram and PCA results. These findings suggest that the genetic characteristics of peanut cultivars are associated with the accumulation levels and coordinated accumulation patterns of three key traits: resveratrol, high oil (fat), and high oleic acid contents. Furthermore, this study may support prioritization for the breeding prioritization of specific phenotypic combinations, such as cultivars featuring high *trans*-piceid and sugar levels, or those combining high *cis*-resveratrol with high oleic acid and oil content.

Based on a comprehensive analysis of resveratrol content, quality indicators, and their correlations across the 42 peanut cultivars, and in adherence to the screening criteria outlined in the Agricultural Industry Standard *Technical Specifications for Evaluation of Peanut Processing Suitability* (NY/T 4283-2023) [39], this study evaluated the suitability of these cultivars for oil extraction, peanut butter production, and protein processing. Screening identified comparatively favorable high-resveratrol germplasms for each application. The Weihua series from Shandong (specifically, Weihua 23) and the Kainong series from Henan (specifically, Kainong 602) were identified as top candidates for oil extraction. For peanut butter production, the Yuhua series (Yuhua 23) and Kainong series (Kainong 58) from Henan were selected as the leading candidates’ cultivars, whereas the Kainong (Kainong 1760) and Yuhua (Yuhua 47) series were identified as the candidate choices for soluble and gel-type protein processing, respectively.

## 4. Conclusions

In conclusion, this study comprehensively mapped the resveratrol profiles and multidimensional quality traits of 42 major peanut cultivars in China. We established that trans-piceid is the predominant resveratrol form and that its accumulation is highly genotype-dependent. A key scientific contribution of this work is the identification of intrinsic correlations between functional phytochemicals (resveratrol derivatives) and primary metabolites (carbohydrates and lipids), which highlights the feasibility of simultaneously selecting for health-promoting compounds and desirable processing traits in multi-trait breeding programs. Ultimately, based on industry standards, we successfully identified seven elite high-resveratrol candidate cultivars tailored for specific industrial applications (e.g., oil extraction and peanut butter production). These findings provide a robust material basis and theoretical reference for the targeted genetic improvement and high-value utilization of peanuts.

## 5. Discussion

This study systematically analyzed the resveratrol content and multidimensional quality indicators in 42 main cultivated peanut varieties in China. Consistent with recent studies on resveratrol [40,41], our results revealed that the trans-glycoside form (trans-piceid) constitutes the predominant and primary storage form of resveratrol in peanut [42]. Significant variations were observed in the total resveratrol, trans-piceid, and cis-/trans-resveratrol contents among the varieties (ranking: Weihua series > Kainong series > Huayu series > Yuhua series > Jihua series > Puhua series > Fuhua series), unlike previous studies that focused on limited local varieties [43]. Significant variation was observed among the seven peanut series, highlighting the influence of genotype on resveratrol accumulation, in line with previous reports on varietal differences in phenolic composition [44].

Furthermore, correlation analysis revealed positive associations between total resveratrol (and trans-piceid) and sucrose, as well as between distinct resveratrol isomers and specific fatty acids (oleic, stearic, and linoleic acids). Although these correlations do not imply direct causality, they suggest an interconnected network between polyphenol biosynthesis and primary carbohydrate/lipid metabolism.

## Figures and Tables

**Table 1:** Sources and names of 42 peanut varieties.

Province of Origin	Soil and Climate	Series Name	Quantity	Variety Name
Shandong	The region features sandy loam soils with low organic matter and a warm temperate semi-humid monsoon climate, marked by seasonal drought and variable precipitation.	Huayu	13	Huayu 9125, Huayu 9124, Huayu 9123, Huayu 9118, Huayu 910, Huayu 616, Huayu 615, Huayu 613, Huayu 50, Huayu 36, Huayu 33, Huayu 25, Huayu 22
		Weihua	4	Weihua 22, Weihua 23, Weihua 25, Weihua 27
Henan	The region features sandy fluvo-aquic soils with moderate fertility and a warm temperate continental monsoon climate, characterized by erratic precipitation and frequent spring droughts.	Kainong	8	Kainong 1715, Kainong 1760, Kainong 58, Kainong 301, Kainong 601, Kainong 602, Kainong 603, Kainong 61
		Yuhua	5	Yuhua 22, Yuhua 23, Yuhua 37, Yuhua 47, Yuhua 9326
		Puhua	4	Puhua 1218, Puhua 1314, Puhua 28, Puhua 76
Hebei	The region features sandy loam soils with low organic matter and a temperate semi-arid monsoon climate, marked by pronounced spring droughts and uneven rainfall distribution.	Jihua	4	Jihua 10, Jihua 13, Jihua 16, Jihua 18
Liaoning	The region features coarse-textured sandy soils with low nutrient content and a temperate semi-humid to semi-arid monsoon climate, characterized by severe spring droughts.	Fuhua	4	Fuhua 22, Fuhua 25, Fuhua 26, Fuhua 27

The names of the peanut varieties were sourced from the China Seed Industry Data Platform. http://202.127.42.47:6010/index.aspx, accessed on 1 October 2025.

**Table 2:** Resveratrol content of different series of peanut varieties (mg/kg).

Peanut Series	Type	Max Value	Min Value	Average	Standard Deviation	Variation Coefficient/%
Jihua	*trans*-Resveratrol	15.36	1.67	5.34	5.79	108.24
	*trans*-Piceid	44.47	19.93	28.71	9.47	32.98
	*cis*-Resveratrol	2.80	ND	0.83	1.16	139.48
	Total resveratrol	46.14	22.26	34.88	9.38	26.90
Weihua	*trans*-Resveratrol	2.97	1.74	2.17	0.48	22.10
	*trans*-Piceid	85.82	32.88	50.82	20.64	40.61
	*cis*-Resveratrol	5.83	ND	1.48	2.52	170.44
	Total resveratrol	88.79	35.00	54.47	20.76	38.12
Kainong	*trans*-Resveratrol	3.26	1.28	2.42	0.64	26.45
	*trans*-Piceid	67.61	13.35	37.88	20.32	53.66
	*cis*-Resveratrol	11.47	ND	3.38	3.96	117.08
	Total resveratrol	76.57	16.60	43.68	20.78	47.58
Yuhua	*trans*-Resveratrol	3.13	ND	1.44	1.27	88.69
	*trans*-Piceid	64.44	16.55	30.56	17.38	56.87
	*cis*-Resveratrol	ND	ND	ND	ND	ND
	Total resveratrol	66.93	16.55	32.00	17.94	56.08
Puhua	*trans*-Resveratrol	2.27	ND	0.83	0.86	103.44
	*trans*-Piceid	31.68	3.04	22.95	11.77	51.30
	*cis*-Resveratrol	5.37	ND	1.77	2.11	119.07
	Total resveratrol	33.06	4.61	25.55	12.10	47.36
Fuhua	*trans*-Resveratrol	1.80	0.53	0.95	0.52	54.56
	*trans*-Piceid	29.76	19.11	24.98	3.93	15.71
	*cis*-Resveratrol	2.04	ND	1.13	0.76	67.69
	Total resveratrol	33.59	20.99	27.06	4.48	16.56
Huayu	*trans*-Resveratrol	23.85	ND	5.95	8.13	136.68
	*trans*-Piceid	57.21	ND	28.36	18.34	64.67
	*cis*-Resveratrol	2.74	ND	0.66	0.93	141.03
	Total resveratrol	60.07	16.33	34.97	13.02	37.22

Note: (ND: Indicates not detected).

**Table 3:** Fatty acid composition of different peanut varieties.

Peanut Series	Fatty Acid Composition	Range of Change (%)	Average	Standard Deviation	Amplitude	Coefficient of Variation %
Huayu	C18:1	37.85–79.08	55.94	14.84	41.23	27.65
	C18:2	4.08–37.13	23.08	22.37	33.05	65.11
	C16:0	6.13–11.72	8.30	22.37	5.59	0.42
	C20:0	0.84–1.32	1.15	22.28	0.48	23.41
	C18:0	1.96–3.10	2.60	21.49	1.14	13.36
Weihua	C18:1	48.61–79.78	71.30	13.12	31.17	18.39
	C18:2	4.43–30.75	11.29	11.24	26.32	99.58
	C16:0	5.11–12.93	8.21	2.90	7.82	35.30
	C20:0	0.97–2.89	1.97	0.71	1.92	35.95
	C18:0	0.98–1.50	1.26	0.19	0.52	14.70
Kainong	C18:1	36.11–79.92	60.08	16.44	43.81	27.37
	C18:2	3.15–47.87	21.81	15.45	44.72	70.83
	C16:0	6.26–12.08	9.14	2.70	5.82	29.57
	C20:0	1.11–1.53	1.43	0.13	0.42	9.17
	C18:0	1.05–1.41	1.26	0.12	0.36	9.79
Yuhua	C18:1	47.21–77.25	54.86	11.36	30.04	20.71
	C18:2	3.3–42.15	27.10	12.82	38.85	47.29
	C16:0	6.61–7.95	7.37	0.51	1.34	6.99
	C20:0	1.06–2.00	1.50	0.35	0.94	23.58
	C18:0	0.74–1.57	1.07	0.31	0.83	28.97
Puhua	C18:1	34.09–77.24	58.81	19.32	43.15	32.85
	C18:2	4.27–46.97	22.07	18.31	42.7	82.97
	C16:0	6.47–11.76	8.99	2.52	5.29	28.00
	C20:0	1.45–1.78	1.62	0.14	0.33	8.77
	C18:0	0.90–1.24	1.13	0.22	0.34	19.71
Fuhua	C18:1	70.26–78.31	74.15	3.90	8.05	5.25
	C18:2	4.66–12.31	8.72	3.60	7.65	41.33
	C16:0	6.29–6.55	6.40	0.10	0.26	1.52
	C20:0	1.29–1.48	1.37	0.07	0.19	5.21
	C18:0	1.24–1.30	1.27	0.02	0.06	1.79
Jihua	C18:1	51.88–76.57	66.59	10.46	15.70	24.69
	C18:2	4.34–36.99	16.40	13.40	81.71	32.65
	C16:0	7.02–7.93	7.35	0.37	5.03	0.91
	C20:0	0.62–1.42	0.97	0.32	32.56	0.8
	C18:0	1.00–1.61	1.42	0.24	17.30	0.61

## Data Availability

The original contributions presented in the study are included in thearticle, further inquiries can be directed to the corresponding author.
